# ‘First, Do No Harm’: The Role of Negative Emotions and Moral Disengagement in Understanding the Relationship Between Workplace Aggression and Misbehavior

**DOI:** 10.3389/fpsyg.2018.00671

**Published:** 2018-05-11

**Authors:** Roberta Fida, Carlo Tramontano, Marinella Paciello, Chiara Guglielmetti, Silvia Gilardi, Tahira M. Probst, Claudio Barbaranelli

**Affiliations:** ^1^Norwich Business School, University of East Anglia, Norwich, United Kingdom; ^2^Centre for Advances in Behavioural Science, Coventry University, Coventry, United Kingdom; ^3^Faculty of Psychology, Università Telematica Internazionale Uninettuno, Rome, Italy; ^4^Dipartimento di Economia, Management e Metodi Quantitativi, Università degli Studi di Milano, Milan, Italy; ^5^Department of Psychology, Washington State University Vancouver, Vancouver, WA, United States; ^6^Department of Psychology, Sapienza Università di Roma, Rome, Italy

**Keywords:** workplace aggression, moral disengagement, discrete negative emotions, misbehavior, health, bullying

## Abstract

Workplace aggression is a critical phenomenon particularly in the healthcare sector, where nurses are especially at risk of bullying and third-party aggression. While workplace aggression has been frequently examined in relation to health problems, less is known about the possible negative impact such aggression may have on the (un)ethical behavior of victims. Our research aims to fill this gap. Drawing on literature on counterproductive work behavior (CWB) and the social-cognitive literature on aggression we investigated in two independent studies (*N*_Study1_ = 439; *N*_Study2_ = 416), the role of negative emotions – in particular anger, fear, and sadness, – and of moral disengagement (MD) in the paths between workplace aggression, CWB and health symptoms. The focus on these relationships is rooted in two reasons. First, misbehavior at work is a pervasive phenomenon worldwide and second, little research has been conducted in the healthcare sector on this type of behavior despite the potential importance of the issue in this context. We empirically tested our hypotheses considering a specific form of workplace aggression in each study: workplace bullying or third-party aggression. Results from the two empirical studies confirm the hypotheses that being target of workplace aggression (bullying or third-party aggression) is not only associated with health symptoms but also with misbehavior. In addition, the results of structural equation modeling attest the importance of examining specific discrete negative emotions and MD for better understanding misbehavior at work. In particular, this research shows for the first time that anger, fear, and sadness, generally aggregated into a single dimension, are indeed differently associated with MD, misbehavior and health symptoms. Specifically, in line with the literature on discrete emotions, while sadness is only associated with health symptoms, anger and fear are related to both health and misbehavior.

## Introduction

Work-related aggression and violence are serious safety and health hazards. According to the European Agency for Safety and Health at Work [EU-OSHA] (2010), one of the highest incidences of workplace violence is found in the healthcare sector (e.g., Pompeii et al., 2013). In this context, nurses are particularly targeted from colleagues as well as patients and their relatives (namely third-party aggression) (e.g., Camerino et al., 2008; Eurofound, 2015). Pompeii et al. (2013) reported that 22–90% of healthcare workers suffer verbal abuse, 12–64% physical threats, and 2–32% physical violence. Within the United States, data from the National Crime Victimization Survey (Harrel, 2011) showed that from 2005 to 2009, while the rate of workplace violence per 1,000 employed persons aged 16 or older was 5.1 for all occupations, this rate raised to 8.1 for nurses.

Workplace aggression is associated with lower psychological wellbeing and life satisfaction, lower levels of self-esteem, higher absenteeism, health problems and burnout (e.g., Camerino et al., 2008; Hills and Joyce, 2013). In particular, physical symptoms such as sleeping problems and headaches have often been reported as consequences of workplace aggression and the emotional distress resulting from it (O’Moore et al., 1998; Quine, 1999; Djurkovic et al., 2004).

**FIGURE 1:**
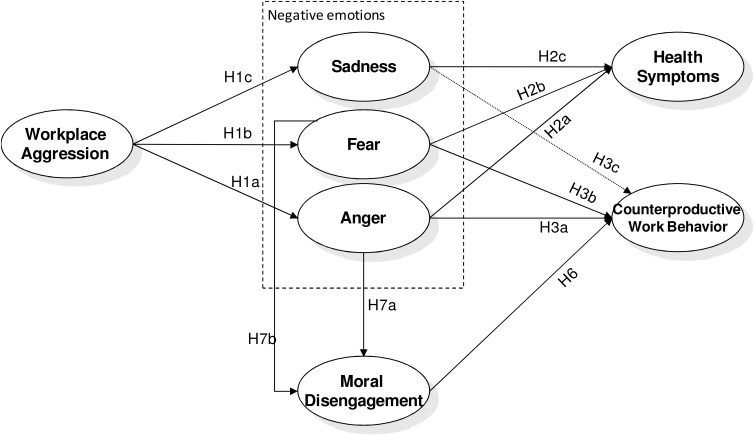
Posited Model: the role of discrete negative emotions and moral disengagement in the relationship between being target of aggression and engaging in counterproductive work behavior.

However, less is known about the possible negative impact such aggression may have on the (un)ethical behavior of victims at work. The aim of this research is to fill this gap. Specifically, we aim to investigate the association of workplace aggression not only with targets’ health but also with their engagement in counterproductive work behavior (CWB). This is an umbrella term referring to aggressive and deviant behavior at work and, more in general, to any act violating the legitimate interests of the organization, and harming it or its stakeholders (Spector and Fox, 2005). The focus on this relationship is rooted in two reasons. First, misbehavior at work is an increasing and costly phenomenon worldwide (e.g., Mount et al., 2006). Second, little research has been conducted in the healthcare sector on this type of behavior despite the potential importance of the issue in this context. In this research, we aim to explore in two independent studies, the role that specific discrete negative emotions (i.e., anger, fear, and sadness) and moral disengagement (MD) may have in explaining the association between being a target of workplace aggression and engaging in this type of misbehavior at work (**Figure 1**).

The hypothesis that targets of aggression may also be perpetrators is not new within the workplace aggression literature (e.g., Andersson and Pearson, 1999; Baron et al., 1999; Hershcovis and Barling, 2010), and the hypothesis that in general job stressors influence CWBs through negative emotions (stressor-emotion model of CWB, Spector and Fox, 2005) and MD (Fida et al., 2015a) is also well established. However, there are no studies to date examining the specific role of frequent mistreatments from either colleagues and supervisor or patients and their relatives. In addition, the discrete emotions perspective has been never adopted to investigate whether the substantive qualities of them may play a specific role beyond their common negative valence. Indeed, as suggested by Kong and Drew (2016, p. 35) studies on emotions and unethical behavior “in which negative emotions are combined into a single category, will produce biased or even misleading results.”

In this research, we focused on three specific discrete emotions: fear, anger, and sadness. We explored them because they are the mostly frequently experienced by targets of aggression (Gerberich et al., 2004; Lanctôt and Guay, 2014). In addition, although these emotions are quite different (Lazarus, 2006) they have been commonly aggregated into a ‘broad’ negative emotions dimension to study the stressors-CWB path. Anger has been also investigated because it is the emotion most often associated with aggressive and antisocial behavior (Anderson and Bushman, 2002). Fear, although generally overlooked in studying the enactment of aggressive behavior at work, has been proven to play a key role as a mediator in the relationship between workplace aggression and other relevant organizational and health-related outcomes (e.g., Rogers and Kelloway, 1997). Furthermore, this emotion is a core variable in explaining aggressive behavior within the literature on defensive aggression (Weinshenker and Siegel, 2002; Simunovic et al., 2013). Finally, sadness has also been included because, although it is an emotion included in the ‘broad’ measure of negative emotions (e.g., Spector and Fox, 2005) differently from anger and fear, it is characterized by lower levels of arousal (Russell, 1980; Posner et al., 2005) and it should be mostly associated with health-related rather than aggressive outcomes (Eisenberg et al., 2001; Kong and Drew, 2016).

An additional contribution of this research also lies in the examination of discrete emotions in relation to MD. Indeed, although some authors suggested the role of negative emotions in increasing the likelihood of the recourse of MD mechanisms (Fida et al., 2015a; Rubio-Garay et al., 2016) no studies, to the best of our knowledge, have examined whether and how the discrete emotions under study may be differently associated with MD. Overall, the examination of the model under study is fundamental to better understand the different paths linking the experience of aggression with engagement in misbehavior at work. This is consistent with one of the most well-known social-cognitive models of aggression (General Aggression Model; Anderson and Bushman, 2002), in which both cognition and emotions are integrated. Our approach is also consistent with the literature on emotions and moral processes highlighting the central role of specific emotions to understand the moral decision-making process (e.g., Haidt, 2001; Hindriks, 2015). Finally, it also in line with Kong and Drew (2016, p. 24) claiming that “collapsing all negative emotions into a single category belies the fact that humans experience a variety of negative emotions, which may have differential effects on disengagement from unethical behavior.”

### Workplace Aggression, CWB and Health: The Role of Negative Emotions

Workplace aggression is a salient source of stress that leads targets to experience a range of negative emotions, especially anger, fear, and sadness (e.g., Lanctôt and Guay, 2014). Within the stressor–emotion model of CWB (Spector and Fox, 2005), rooted in the frustration–aggression hypothesis and the Lazarus’ model of stress (Lazarus, 2006), negative emotions play a pivotal role for explaining CWB. In particular, these behaviors are conceptualized as an aversive response to negative emotions experienced at work. Overall, several studies clearly confirmed this model and showed that when employees perceive stressors at work (i.e., workload, organizational constraints, and interpersonal conflicts) they possibly experience a broad range of negative emotions, like anger, fear, and sadness that increase the likelihood for them of engaging in CWB (e.g., Bruk-Lee and Spector, 2006).

In framing this model in the frustration-aggression hypothesis, Spector and Fox seem to suggest that the emotion mostly associated with CWB is anger. Indeed, the broader literature on aggression suggests the causal role of anger in triggering aggressive conduct. This is also consistent with findings from Rodell and Judge (2009), showing that anger is the emotion mainly involved in the relationship between hindrance stressors and the engagement in CWB. Anger is experienced when individuals believe their own rights or shared rules have been violated (Power and Dalgleish, 2008), and when they have suffered an attack or damage to their own identity or role that is considered unfair and unacceptable (Lazarus, 2006). Anger does not only influence individuals’ behavior but also their health. In particular, anger can have a direct impact on cardiovascular diseases due to an excessive liberation of stress hormones. In addition, it can also be associated with somatic symptoms (e.g., Consedine and Moskowitz, 2007) and with the adoption of an unhealthy lifestyle (Staicu and Cuţov, 2010).

Literature on emotions has also highlighted that fear can be another high-arousal emotion associated with engaging in aggressive behavior (Blanchard et al., 2001; Simunovic et al., 2013; Kong and Drew, 2016). However, this hypothesis has been overlooked in relation to organizational misconduct and in this study we aim to explore the role of fear on CWB above and beyond anger. Fear is one of the basic emotions activated in alarm situations that individuals consider as potentially risky for their own life goals, for example their survival (Öhman, 2008). Fear shares with anger not only the negative emotional valence but also the fact that there is a perception of damage (that is actual in the case of anger, rather than mainly dreaded in the case of fear). However, in contrast with anger, fear is associated with a perceived lower power to face the negative events (Lerner and Keltner, 2001). Indeed, when feeling fear, an individual’s agency tends to be quite low and events are perceived as uncontrollable and regulated by external causes (Lerner and Keltner, 2001). Hence, it is likely that employees experiencing fear may consider themselves as lacking the resources needed to exert control over the events. Previous research attests that, when individuals experience this emotion, they tend to judge more pessimistically the progression of the events across situations, above and beyond the actual event that activated it.

Fear has traditionally been associated with escape behavior that allows individuals to distance themselves from threatening events that are potentially dangerous (Lazarus, 2006). However, this emotion has been also associated with aggressive behaviors especially when individuals perceived a threat and when this behavior is motivated by the need to defend oneself (Simunovic et al., 2013). Literature in the organizational context showed that a frequent experience of fear may result in a variety of negative consequences. It is associated with mental health and physical symptoms (Rogers and Kelloway, 1997), and with withdrawal conduct such as job neglect, absenteeism, and job turnover (e.g., Barling, 1996).

Sadness is another discrete emotion experienced by targets of aggression also included in the negative emotions measure generally adopted when examining stressors-CWB path. It has a negative valence, but in contrast with anger and fear, it is associated with lower arousal. Sadness is activated when there is a perception of loss or failure of something valued (Lazarus, 2006) in relation to which individuals feel powerless. When people experience this type of emotion they tend to react, for example, with discouragement, resignation or withdrawal in an attempt to elaborate their loss and to restore their psychological and social equilibrium (Horwitz and Wakefield, 2007). Indeed, the main function of sadness is to increase self-focus and allow individuals to adjust themselves to the new and undesired situation by accepting what cannot be modified (the loss) and by finding new adapting strategies (Horwitz and Wakefield, 2007; Power and Dalgleish, 2008). Sadness also has a social function, being associated with seeking help and support from others (Izard, 1993).

Sadness is associated with several health-related issues, such as reduced energy (Consedine and Moskowitz, 2007), heart problems (Gullette et al., 1997), and somatic symptoms (Consedine et al., 2006). The only two studies examining this emotion in relation to misbehavior showed non-significant results even in relation to withdrawal (Khan et al., 2013; Bauer and Spector, 2015).

Overall, according to the literature described above we hypothesize that (see **Figure 1**): workplace aggression is positively associated with anger (H1a), fear (H1b), and sadness (H1c) that in their turn are associated with health symptoms (H2a, H2b, and H2c). We also hypothesize that only anger and fear, but not sadness, are associated with CWB (H3a, H3b, and H3c, respectively). Hence, we hypothesize that the relationship between being target of aggression and health symptoms is mediated by anger, fear, and sadness (H4a, H4b, and H4c, respectively), and the relationship between being target of aggression and CWB is mediated only by anger and fear (H5a and H5b).

### Workplace Aggression, Negative Emotions and CWB: The Role of Moral Disengagement

Notwithstanding the pivotal role of affective experience for understanding aggression at work, according to the social-cognitive perspective (Anderson and Bushman, 2002), it is also important taking into account the role of cognitive dimensions that could mediate the relationship between emotions and behavior. Within the broader literature, MD has been studied as a relevant cognitive dimension that helps to better understand why any individual may misbehave (e.g., Fida et al., 2015a). MD is a social–cognitive construct referring to a set of processes by which individuals can justify and legitimate their misbehavior (Bandura, 1991). According to Bandura, individuals have moral standards guiding their actions and generally tend to engage in behavior that brings them self-worth and satisfaction, while refraining from conduct that violates those standards and results in self-condemnation, guilt, and shame. However, Bandura (2016) also highlighted that these internal standards are not constantly active. Indeed, people may possibly resort to cognitive mechanisms aimed at temporarily silencing their moral standards, allowing them freely to engage in conduct they would generally consider reprehensible, without perceiving (or at least reducing) any inconsistencies between their internal moral system and their actual behavior. According to Hindriks (2015) MD is a rationalization process likely to be activated to address a cognitive dissonance between individuals’ standards and the envisaged action not in line with them.

MD can operate at different levels: redefining the behavior itself, altering the perception of its consequences, obscuring the agentic role of the perpetrator, and depicting the victim as responsible. Specifically, at the behavior level, the re-construction of the misconduct can occur through several mechanisms: *moral justification* – i.e., justifying a misconduct as serving a higher moral good or a potential benefit for others (e.g., “It is alright to fly off the handle to protect your friends”); *euphemistic labeling* – i.e., depicting a misconduct using mild language that masks its reprehensive nature (e.g., “Slapping and shoving someone is just a way of joking”); and *advantageous comparison* – i.e., reducing the perception of an act as misbehavior by comparing it with more flagrant misconduct (e.g., “Damaging some property is no big deal when you consider that others are beating people up”). At the consequence level, it can occur through *disregarding or distortion of consequences* – i.e., minimizing or altering the actual negative consequences of misbehavior (e.g., “It is not serious to tell small lies because they don’t hurt anybody”). At the agentic level, it can occur through the following: *displacement of responsibility* – i.e., considering one’s own misbehavior as dictated by a superior authority or social pressure (e.g., “If youth are living under bad conditions in their neighborhood they cannot be blamed for behaving aggressively”); and *diffusion of responsibility* – i.e., diminishing one’s own responsibility for misbehavior by considering the harm produced as resulting from a collective action (e.g., “A member of a group should not be blamed for trouble the group causes”). At the victim level, it can occur through the following: *attribution of blame* – i.e., attributing responsibility for the suffered misconduct to the victim (e.g., “If people fight and misbehave in school or at work it is their teacher’s/superior’s fault”); and *dehumanization* – i.e., draining the victim of their human characteristics and considering them as sub-human (e.g., “Some people deserve to be treated like animals”). All of these mechanisms serve the same end: allowing individuals to morally disengage from their own actions and, as a result, legitimizing their aggressive and deviant behavior while keeping the same moral standards (Bandura, 2016).

Within the organizational literature, Moore et al. (2012) highlighted the added value of MD in explaining unethical organizational behavior, above and beyond morally related individual traits, moral reasoning, and dispositional moral emotions. Additionally, Moore and Gino (2013) proposed that MD should be considered as a disruptor of the so-called moral compass – i.e., the internal mechanism stabilizing and orientating individuals’ behavior to agree with their internal moral standards. Fida et al. (2015a) examined the role of MD in understanding why workers, under stressful circumstances, may behave counterproductively. Specifically, they found support for its mediational role in the relationship between negative emotions associated with organizational stressors and CWB. Overall, although there has been an increasing interest in the investigation of MD within organizational settings research in this area can still be considered limited (Johnson and Buckley, 2014).

Since misbehavior at work happens within a system of social and organizational norms rather than in a vacuum, it should not be considered as exclusively impulsive. In fact, misconduct at work may need to be planned and anticipated, at least in some cases. For example, a verbal aggression toward a colleague may be a way to release negative emotions such as frustration and anger. However, calling in sick when this is not the case it is more likely to require some forms of justification cognitive process. Thus, although this type of misconduct may still be rooted in negative emotional activation, in order to be acted upon it requires the individual to reframe the misbehavior, making it a viable option. Similarly, when considering clinical misconduct such as administering a different drug dose without consulting a physician, it is likely that justification cognitive processes may also play a role above and beyond the affective ones. Hence, we hypothesize that MD is positively associated with CWB (H6).

In addition, within the literature on unethical decision-making process it has been suggested the importance of considering stress and emotions to understand what can trigger rule breaking behaviors (Selart and Johansen, 2011). Furthermore, Kong and Drew (2016), referring to the resource depletion theory, highlighted that when individuals are emotionally activated and their resources are diminished it is more likely the engagement in selfish and antisocial behavior due to a weakened moral self-regulation system (e.g., Barnes et al., 2011; Christian and Ellis, 2011).

In line with this, recently Fida et al. (2015a) showed that the broad negative emotions dimension is an antecedent of MD which in its turn mediates the relationship between emotions and CWB. However, as previously underlined, negative emotions should be examined separately to better understand different processes that may result in similar outcomes. Rubio-Garay et al. (2016) have already supported the specific contribution of anger on MD for understanding aggressive behavior. Indeed, anger leads individuals to maintain an aggressive intention over time and to have a hostile bias in interpreting neutral and ambiguous situations, and to attribute externally the causes of their misbehavior (Anderson and Bushman, 2002). We believe that also fear can be related to MD. In line with literature, when individuals experience fear they could activate self-serving and egoistic patterns of reactions when facing threatening situations (Lerner and Keltner, 2001). In addition, this emotion promotes egoistic attitudes in acute stressful situations and interferes with the tendency to empathize and to engage in prosocial acts (Starcke et al., 2011).

Hence we hypothesize that both anger and fear will be related to MD (respectively, H7a and H7b), while sadness will not (H7c). Finally, consistently with the overall set of hypotheses we expect that: workplace aggression is indirectly associated with CWB through anger and MD (H8a), and fear and MD (H8b).

### Overview of the Present Research

In this research, we aim to understand better the role of negative emotions – in particular of anger, fear, and sadness – and of MD in the relationship between being a target of workplace aggression and engaging in CWB by testing the posited models in two independent studies. In each study we considered a specific form of workplace aggression, respectively, bullying and third-party aggression, and specific forms of CWB, consistent with the type of aggression under study. The rationale for this is to strengthen the validity of our results and their generalizability (Sackett and Larson, 1990). Indeed, the examination of the posited models in two different samples and considering two different types of aggression, whose source is in one case internal to the organization and in the other external, is pivotal to clarify the role of emotions and MD in relation to different frustrating and stressful aggressive events.

## Study 1: Bullying and CWB

In this study, we focus on workplace bullying, health symptoms and on both interpersonal and organizational CWB. Bullying has been identified as a widespread phenomenon in healthcare work environments (e.g., Quine, 2001). The prevalence rates of bullying experienced by nurses vary widely in the literature, with studies reporting, for example, rates of 7.6% (Einarsen and Skogstad, 1996), 30% (Farrell, 1999), 27.3% (Johnson and Rea, 2009), and 44% (Quine, 2001). Recent organizational literature (e.g., Branch et al., 2013) highlighted the regrettable incidence of this specific form of aggression, reporting that between 10 and 18% of workers, both in Europe and North America, have been victims of bullying.

Bullying may include both interpersonal forms of aggression, ranging from spreading of gossip to physical threats and harassment, and work-related negative acts, such as the victim being ordered to do work below their level of competence (Einarsen and Raknes, 1997). Consistent with this distinction, when designing the study, we measured both CWB targeting individuals and those targeting the organization as a whole.

In order to highlight better the added value of examining the aggression-CWB relationship considering the three discrete emotions, we prior tested a model (Model 1 – General negative emotion) in which negative emotions were operationalized in line with the stressor-emotion model (i.e., one dimension comprising a broader range of negative emotions) and then examined the hypothesized model with anger, fear, and sadness separately (Model 2 – Discrete negative emotions).

### Participants and Procedure

Participants were nurses selected using a convenience sampling procedure. An anonymous paper-and-pencil questionnaire (in a blank envelope) was distributed to nurses by research assistants. Specifically, data were collected by nursing students attending a master program in nursing management as part of their coursework. In particular, they were asked to recruit five nurses they knew who worked in public or private healthcare organization. As a consequence, the nurses included in the final sample are not explicitly clustered in specific organizations. The nurses were expected to return the blank envelope containing the completed questionnaire by the following week. Before starting, the research assistants explained that responses would be kept confidential and asked them to sign a written informant consent. Participation was voluntary and no rewards were provided. The research protocol was approved by the ethical board of the department to which the first author was affiliated when the study was designed.

The final sample comprised 439 nurses (75% women) with a mean age of 39 years (*SD* = 8). They had been working as nurses for an average of 17 years (*SD* = 10) and in their current organizations for an average of 13 years (*SD* = 10). About half of the nurses worked in medical/surgical units (52%) and the majority of the sample was employed in public hospitals (87%), with a permanent contract (83%), working full-time (80%) for 7 h per day on average (*SD* = 1). Our sample is in line with the national data in relation to gender (76%; Mangiacavalli, 2017) and it seems slightly younger in terms of age and job tenure (47.70 and 19.53 years, respectively; Del Vecchio, 2016). Given the convenience sampling procedure it was not possible to keep track of the participation response rata.

### Measures

#### Bullying

Bullying was measured by the 11-item Negative Acts Questionnaire (Einarsen and Raknes, 1997). Participants were asked to indicate their frequency of exposure to various negative acts in their workplace during the past 6 months. Response options were presented in a five-point format ranging from *never or almost never* to *once a week or more*. This scale provided two scores for work-related bullying (five items – e.g., *imposing unreasonable deadlines*) and personal bullying (six items – e.g., *threatened with physical abuse*). Preliminary confirmatory factor analysis (CFA) supported the factor structure of the scale [χ^2^(43) = 105.74, *p* < 0.01; *CFI* = 0.91; *RMSEA* = 0.058 (*90% C.I.* = 0.044-0.072), *p* = 0.17; *SRMR* = 0.051].

#### Negative Emotions

Negative emotions were measured by 15 emotions included in the Job-Related Affective Well-being Scale (Van Katwyk et al., 2000). Respondents were asked how often they had experienced each emotion at work during the 30 days immediately preceding the questionnaire. Response options were presented in a five-point format ranging from *almost never* to *extremely often or always*. Preliminary CFA confirmed the factor structure of the scale [χ^2^(86) = 313.52, *p* < 0.01; *CFI* = 0.91; *RMSEA* = 0.078 (*90% C.I.* = 0.069-0.087), *p* < 0.001; *SRMR* = 0.056]. When emotions were considered as discrete, ‘angry,’ ‘irritated,’ ‘furious,’ and ‘frustrated’ were posited as indicators of anger; ‘frightened,’ ‘anxious,’ and ‘intimidated’ indicators of fear; and ‘depressed,’ ‘discouraged’ and ‘miserable’ indicators of sadness. Preliminary CFA confirmed the tenability of this model [χ^2^(21) = 76.95, *p* < 0.01; *CFI* = 0.96; *RMSEA* = 0.078 (*90% C.I.* = 0.060-0.097), *p* < 0.01; *SRMR* = 0.042].

#### Moral Disengagement

Moral disengagement was assessed by the 18-item Nurse Moral Disengagement scale (Fida et al., 2015b). Participants were asked to rate their level of agreement with statements reflecting MD mechanisms in their work activities (e.g., ‘*To be rude to a very demanding patient is not serious if the workload is very heavy*’). Response options were presented in a five-point format ranging from *agree not at all* to *completely agree*. Preliminary CFA confirmed the factor structure of the scale [χ^2^(135) = 347.37, *p* < 0.01; *CFI* = 0.95; *RMSEA* = 0.060 (*90% C.I.* = 0.052-0.068), *p* < 0.05; WRMR = 1.14].

#### Counterproductive Workplace Behavior

Counterproductive workplace behavior was measured by 17 items derived from the CWB Checklist (Spector et al., 2006). Participants were asked to indicate how often they engaged in each of the listed behaviors in their present job. Response options were presented in a five-point format ranging from *never* to *every day*. The scale provided two scores for behaviors that targeted people in the organization (CWB-P) (nine items – e.g., ‘*insulted someone about his or her job performance*’) and behaviors that targeted the organization as a whole (CWB-O) (eight items – e.g., ‘*stole something belonging to an employer*’). Preliminary CFA confirmed the factor structure of the scale [χ^2^(118) = 329.53, *p* < 0.01; *CFI* = 0.96; *RMSEA* = 0.064 (*90% C.I.* = 0.056-0.073), *p* < 0.01; WRMR = 1.20].

#### Health Symptoms

Health symptoms were assessed by six items derived from the Physical Symptoms Inventory (Spector and Jex, 1998). Participants were asked to rate how frequently they had experienced each of the listed symptoms (e.g., ‘headache and concentration difficulties’) during the 30 days immediately preceding the questionnaire. Response options were presented in a five-point format ranging from *not at all* to *every day*. Preliminary CFA confirmed the factor structure of the scale [χ^2^(8) = 10.52, *p* = 0.23; *CFI* = 1.00; *RMSEA* = 0.027 (*90% C.I.* = 0.000-0.066), *p* = 0.80; *SRMR* = 0.016].

### Data Analysis

Before examining the posited structural models, the adequacy of the corresponding measurement model was tested through a confirmatory factorial approach (Bollen, 1989). Then, following Harman’s (1976) recommendation we checked for common method bias by comparing, through the chi-square difference test, the measurement model with an alternative model in which all the items loaded into a single latent factor. Since the study variables tended toward negative skewness and excessive kurtosis (see **Table 1**), robust maximum likelihood (MLR) estimates were used. Workplace bullying, negative emotions (for Model 1- General negative emotion) MD, health symptoms and CWB were measured by parcels (i.e., the average of several items measuring the construct) as indicators of latent variables (Coffman and MacCallum, 2005).

**Table 1:** Study 1: descriptive statistics of the study variables.

	*M*	*SD*	Skewness	Kurtosis	Cronbach’s alpha	2	3	3a	3b	3c	4	5	6	7
(1) Work-related bullying	1.73	0.69	1.50	3.30	0.74	0.53^∗∗^	0.47^∗∗^	0.44^∗∗^	0.30^∗∗^	0.41^∗∗^	0.16^∗∗^	0.07	0.06	0.37^∗∗^
(2) Personal bullying	1.30	0.46	2.99	14.80	0.73	-	0.38^∗∗^	0.36^∗∗^	0.29^∗∗^	0.32^∗∗^	0.15^∗∗^	0.02	0.05	0.30^∗∗^
(3) Negative emotions	2.30	0.73	0.75	0.46	0.91		–	0.85^∗∗^	0.78^∗∗^	0.90^∗∗^	0.23^∗∗^	0.16^∗∗^	0.20^∗∗^	0.46^∗∗^
(3a) Anger	2.52	0.88	0.43	-0.26	0.82			–	0.52^∗∗^	0.68^∗∗^	0.16^∗∗^	0.14^∗∗^	0.22^∗∗^	0.37^∗∗^
(3b) Fear	2.09	0.85	0.72	0.24	0.76				-	0.63^∗∗^	0.23^∗∗^	0.10^∗^	0.06	0.34^∗∗^
(3c) Sadness	2.15	0.93	0.87	0.50	0.79					–	0.15^∗∗^	0.12^∗^	0.17^∗∗^	0.45^∗∗^
(4) Moral disengagement	1.33	0.35	2.19	7.24	0.84						–	0.33^∗∗^	0.30^∗∗^	0.09
(5) CWB-O	1.33	0.40	2.27	6.88	0.79							–	0.68^∗∗^	-0.01
(6) CWB-P	1.21	0.35	3.61	18.92	0.86								–	-0.00
(7) Health symptoms	2.73	0.70	-0.31	-0.49	0.82									–


**CWB-P, interpersonal counterproductive work behavior; CWB-O, organizational counterproductive work behavior. ^∗^p < 0.05, ^∗∗^p < 0.01.**

When examining the model in **Figure 1** (Model 2 – Discrete negative emotions), to explore the specific role of anger, fear, and sadness we identify their unique variances by following Bentler’s non-standard structural equation model approach (1990). First, we computed each discrete emotion by averaging their corresponding indicators. Then, we defined the latent variable *negative emotions*, capturing what these three discrete emotions have in common, and finally we used their corresponding uniqueness in the structural model. Specifically, workplace bullying has been defined as the independent variable influencing the uniqueness of the three discrete emotions that in turn were specified as independent variables influencing health symptoms, MD, and CWB-O and CWB-P. Consistent with Bentler (1990), the specific effects in our non-standard model reflect the contribution of workplace bullying to what is unique about each one of the three discrete emotions, as well as the contribution of what is unique to these three emotions on health symptoms, MD, and CWB-O and CWB-P. Overall, this type of model allows for isolating the uniqueness of the three discrete emotions – namely what characterized each of them above and beyond what they share with the others as reflecting the overarching ‘negative emotions’ factor – and for answering questions regarding the specific effects.

The indirect effect test implemented in Mplus 8.1 was used to examine the hypothesized indirect associations. Furthermore, gender and job tenure have been included as covariates as it is plausible to hypothesize that they can affect each of the study variables.

### Results

#### Descriptive Statistics

Descriptive statistics are presented in **Table 1**. These show that all variables had good reliability. Both bullying dimensions significantly correlated with the broad negative emotions dimension, the three discrete emotions, MD and health symptoms but not with neither CWB-O nor CWB-P. The negative emotions dimension as well as the three discrete emotions correlated with all study variables with the only exception of the non-significant correlation between fear and CWB-P. MD significantly correlated with all study variables with the only exception of health symptoms. Both CWBs did not correlate with health symptoms. Finally, CWB dimensions significantly correlated with each other.

##### Model 1 – General negative emotion

The measurement model resulted in a good fit: χ^2^(104) = 148.94, *p* < 0.01; *CFI* = 0.99; *RMSEA* = 0.031 (*90% C.I.* = 0.019-0.042), *p* = 1.00; *SRMR* = 0.033. Results of the one-factor model showed a poor fit to the data: χ^2^(119) = 2,286.65, *p* < 0.001, *CFI* = 0.37, *RMSEA* = 0.204 (*90% C.I.* = 0.197-0.211), *p* = 0.001; *SRMR* = 0.206, attesting to the discriminant validity of the measures and the absence of common method bias. This result was also confirmed by the significant chi-square difference test between the two models (*p* < 0.001).

**FIGURE 2:**
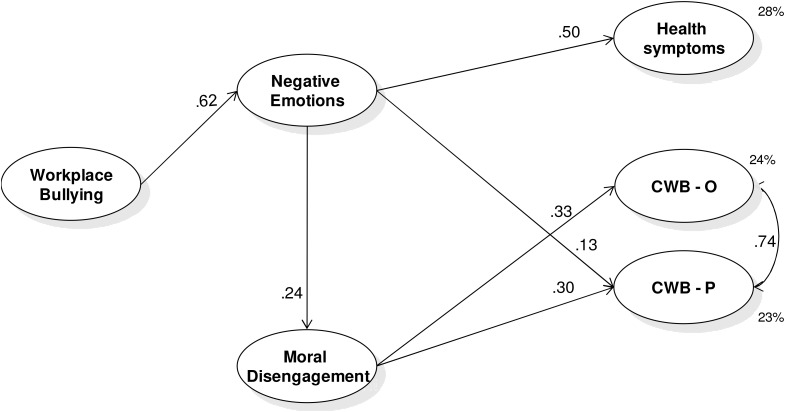
Study 1: results of the posited Model 1 – General negative emotion. All variables are latent variables measured by their indicators as described in the text. Completely standardized robust maximum likelihood parameter estimates. All coefficients reported are significant for *p* < 0.05. The results of the role of the covariates and the non-significant hypothesized paths have not been reported in the figure, but they are discussed in the text. CWB-O, organizational counterproductive work behavior; CWB-*P*, interpersonal counterproductive work behavior.

Model 1 resulted in a good fit: χ^2^(*df* = 131) = 200.26, *p* < 0.01; *RMSEA* = 0.035 (*90% C.I.* = 0.025-0.044), *p* = 1.00; *CFI* = 0.98; *SRMR* = 0.039. Findings, presented in **Figure 2**, showed that bullying was positively associated with negative emotions (H1) that in their turn were positively associated with health symptoms (H2) and only with CWB-P (H3). Consistent with H7, negative emotions were positively associated with MD that in its turn was associated with both CWB-P and CWB-O (H6). Furthermore, the test of the indirect effects confirmed that bullying was associated with health symptoms and CWB-P through negative emotions (H4: β = 0.31, Bootstrap *95% C.I.* = 0.23-0.41, H5: β = 0.08, Bootstrap *95% C.I.* = 0.02-0.17). In addition, results also confirmed that bullying was associated with both CWB-O and CWB-P through negative emotions and MD (H8, β = 0.05, Bootstrap *95% C.I.* = 0.02-0.09; β = 0.05, Bootstrap *95% C.I.* = 0.01 -0.09, respectively). The findings also highlighted significant patterns for covariates. In particular, men scored higher in CWB-P (β = -0.26) and lower in health symptoms (β = 0.16). Employees with shorter job tenure scored lower in negative emotions (β = -0.11), MD (β = -0.45), and CWB-O (β = -0.15).

##### Model 2 – Discrete negative emotions

The measurement model resulted in a good fit: χ^2^(104) = 153.30, *p* < 0.01; *CFI* = 0.98; *RMSEA* = 0.033 (*90% C.I.* = 0.021-0.044), *p* = 1.00; *SRMR* = 0.035. Results of the one-factor model showed a poor fit to the data: χ^2^(119) = 1,912.47, *p* < 0.001, *CFI* = 0.40, *RMSEA* = 0.185 (90% CI = 0.178-0.193), *p* = 0.001; *SRMR* = 0.182, attesting to the discriminant validity of the measures and the absence of common method bias. This result is also confirmed by the significant chi-square difference test between the two models (*p* < 0.001).

**FIGURE 3:**
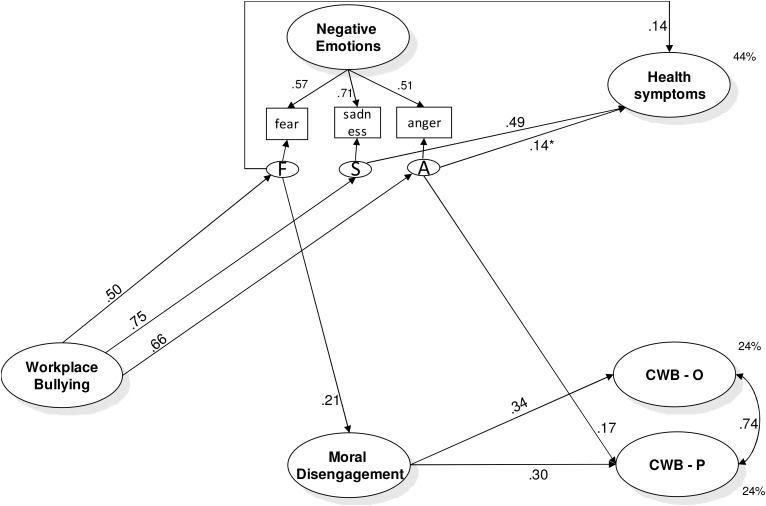
Study 1: results of the posited Model 2- Discrete negative emotions. All variables are latent variables measured by their indicators as described in the text. Completely standardized robust maximum likelihood parameter estimates. All coefficients reported are significant for *p* < 0.05. The results of the role of the covariates and the non-significant hypothesized paths have not been reported in the figure, but they are discussed in the text. F, residual variance of Fear, that is, what Fear does not share with Anger and Sadness; S, residual variance of Sadness, that is, what Sadness does not share with Anger and Fear; A, residual variance of Anger, that is, what Anger does not share with Fear and Sadness; CWB-O, organizational counterproductive work behavior; CWB-*P*, interpersonal counterproductive work behavior. ^∗^*p* < 0.10.

Model 2 resulted in a good fit: χ^2^(128) = 181.87, *p* < 0.01; *CFI* = 0.98; *RMSEA* = 0.031 (*90% C.I.* = 0.020-0.041), *p* = 1.00; *SRMR* = 0.039. Findings, presented in **Figure 3**, showed that bullying was positively associated with all three of the discrete emotions considered (H1a, H1b, and H1c) that in their turn were associated with health symptoms (H2a, H2b, and H2c), although anger only marginally. H3 and H7 were confirmed only partially. In particular, as expected sadness was not associated with either CWB-O or CWB-P (H3c). Anger was associated only with CWB-P but not with CWB-O (H3a) and fear with none of them (H3b). In addition, only fear but not anger was associated with MD (H7a and H7b). Finally, in line with the hypotheses MD was associated with both CWB-O and CWB-P (H6). The test of indirect effects confirmed that workplace bullying was associated with health symptoms through fear (H4b, β = 0.07, *p* < 0.05) and sadness (H4c, β = 0.37, *p* < 0.001), with CWB-P through anger (H5a, β = 0.11, *p* < 0.05), and with both CWB-O and CWB-P through fear and MD (H8b, β = 0.04, *p* < 0.01, β = 0.03, *p* < 0.05, respectively). The findings also highlight significant patterns for covariates. In particular, men scored higher in CWB-O (β = -0.22) and CWB-P (β = -0.27), and lower in health symptoms (β = 0.17). Employees with shorter job tenure scored lower in MD (β = 0.16) and CWB-O (β = -0.15).

### Discussion

Results of this study show that being a target of bullying at work is not only associated with health-related problems but also with misbehaving. In particular, results from Model 1 confirm the hypotheses that the negative emotions experienced when being target of bullying at work are associated with CWB directly, in the case of CWB-P, and both directly and indirectly, through MD, in the case of CWB-O. However, results from Model 2 showed that the association of negative emotions with health symptoms and CWB is more complex when examining anger, fear, and sadness separately. Indeed, while sadness was exclusively associated with health symptoms, anger and fear, besides being associated with health symptoms, were also associated with CWB, although in a different way. Indeed, anger was associated with CWB-P directly, and fear with both CWBs through MD. Overall, these results shows that the experience of anger when being target of bullying may increase the likelihood of the target of misbehaving. Similarly, results showed that when targets experience fear they may also engage in misbehavior at work, targeting both the organization and the individuals, although only through the activation of MD. This result is in line with studies that have proven the role of fear in self-serving and egoistic reasoning under stressful conditions (Starcke et al., 2011), and of those on the role of MD as an important antecedent of aggressive and deviant behavior (Bandura, 2016). However, the findings also suggest that for some types of CWB, MD may not be always needed and the anger activation, associated with being a target of workplace bullying, may facilitate an impulsive form of aggression.

## Study 2: Third Party Aggression and Misbehavior

The aim of this study is to cross-validate the posited models in a different sample considering different variables. In particular, instead of bullying as source of aggression we included third-party aggression. In addition, given the specific source of the aggression under study, along with CWB-P we considered misbehavior in the clinical practice rather than CWB-O. Aggression against healthcare staff from patients and their relatives has also been recognized as one of the most prevalent forms of work-related violence. Specifically, the review by Hills and Joyce (2013) highlighted that the percentage of healthcare workers subjected to aggression from patients ranged from 10 to 95% and those subjected to aggression from patients’ relatives ranged from 20 to 40%. In Europe, results from the NEXT study implemented in 10 countries, found that 22% of nurses were exposed to this form of aggression (Estryn-Behar et al., 2008). Unfortunately, it is likely that these percentages underestimate the true extent of such aggression, since healthcare workers may often consider a limited number of physical assaults and a low level of verbal aggression as ‘part of the job,’ or may perceive the reporting of such incidents as undermining their professional standing and reputation. Similarly to Study 1, we also tested a model in which negative emotions were operationalized in line with the stressor-emotion model.

### Method

#### Participants and Procedure

Research was conducted in six hospitals using a convenience sampling method. Having obtained approval from the hospitals’ managers, an anonymous paper-and-pencil questionnaire (in a blank envelope) was distributed to nurses by research assistants with the support of nurse coordinators. Before starting, the research assistants explained that responses would be kept confidential and that the research was not commissioned by the hospital for which they worked. In addition, the research assistants asked each participant to read and sign a written informant consent. As in Study 1, participation was voluntary and no rewards were provided. The research protocol was approved by the ethical board of the department to which the first author was affiliated when the study was designed. Nurses working in the psychiatric ward were not included in the sampling procedure, since aggression from patients in this unit can be specifically due to their clinical condition being a manifestation of their diagnosis. Thus, it is expected that since nurses working in these types of units may consider being a target of aggression more likely than nurses working in different types of units, their affective and cognitive correlates may be different. The sample comprised 416 nurses (56.7% females) across the six hospitals, with a mean sample size of 69 nurses per hospital, ranging from 26 to 88. Because the exact number of nurses employed in each hospital was not obtained, we were unable to compute the response rates. Participants had a mean age of 42 (*SD* = 9), worked on average 7 h per day and had an average job tenure of 13 years (*SD* = 10). One hundred and forty-two nurses (34%) worked in medical wards, 117 (28%) in emergency rooms, 84 (20%) in surgical wards and 61 (15%) in critical wards; 12 (3%) did not report this information. In this case our sample seems to be more gender balanced and younger in terms of age and job tenure than the national nursing population (Del Vecchio, 2016; Mangiacavalli, 2017). Given the convenience sampling procedure it was not possible to keep track of the participation response rata.

#### Measures

##### Third-party aggression

Third-party aggression was measured by three items that asked how often in the last 12 months the participant reports to have been exposed to (1) physical aggression, (2) threats of physical aggression, (3) verbal aggression by patients and their relatives. These forms of aggression refer to those indicated by European Agency for Safety and Health at Work [EU-OSHA] (2010) report as “Type 2 Consumer-related violence: consumer/clients/patients (and family) violence against staff, vicarious trauma to staff, staff violence to clients/consumers” (Bowie et al., 2012, p. 3) also adopted in the Violent Incident Form by Arnetz (1998). Compared to the Arnetz’s version the response scale has been changed: from a three-point scale (no, never; yes, once or twice; yes, several times) to a five-point scale which includes a reference to a concrete time unit (Never; Few times; About 1 time a month; About 1 time a Week; Daily). Preliminary CFA showed that loadings ranged from 0.68 to 79.

##### Negative emotions, MD and health symptoms

Negative emotions, MD and health symptoms were measured as in Study 1. Preliminary CFA provided support for the factor structure of all the scales [negative emotions: χ^2^(84) = 308.934; *p* < 0.001; *RMSEA* = 0.080 (*90% C.I.* = 0.071–0.090), *p* < 0.001; *CFI* = 0.90; *SRMR* = 0.061; MD: χ^2^(130) = 369.81; *p* < 0.001; *RMSEA* = 0.067 (*90% C.I.* = 0.059–0.075), *p* < 0.001; *CFI* = 0.89; *SRMR* = 0.062; Health Symptoms: χ^2^(8) = 16.43; *p* < 0.001; *RMSEA* = 0.050 (*90% C.I.* = 0.012–0.085), *p* = 0.44; *CFI* = 0.99; *SRMR* = 0.022].

##### Interpersonal CWB (CWB-P)

Interpersonal CWB (CWB-P) was measured by four items (e.g., ‘*Threatened someone at work, but not physically*’) from the CWB Checklist (Spector et al., 2006). Participants were asked to indicate how often they engaged in each of the listed behaviors in their present job. Response options were presented in a five-point Likert format from *never* to *always*.

##### Clinical misbehavior

Clinical misbehavior was measured by five items developed for the purpose of the present study by adapting the Nursing Counterproductive Work Behavior Scale (Sili et al., 2014; **see Appendix A** for the items). Participants were asked to indicate how often they engaged in each of the listed behaviors in their present job. Response options were presented in a five-point Likert format from *never* to *always*. Since these items have been developed for this study, a preliminary confirmatory factor analysis was implemented and provided support for its factor structure: χ^2^(5) = 10.681; *p* = 0.06; *RMSEA* = 0.052 (*90% C.I.* = 0.000–0.096), *p* = 0.40; *CFI* = 1.00; *WRMR* = 0.424. In addition, the CFA considering both interpersonal CWB and clinical misbehavior also supported the factor structure [χ^2^(26) = 112.09; *p* = 0.06; *RMSEA* = 0.089 (*90% C.I.* = 0.073–0.107), *p* < 0.001; *CFI* = 0.99; *WRMR* = 1.694].

#### Data Analysis

Study 2 follows the same analytical approach as that adopted for Study 1. Given the hierarchical structure of our data (nurses were nested within six different hospitals and potentially interacted with the same patients), data were analyzed using the procedure ‘type is complex.’ In addition, considering that each hospital might have different norms regarding the management of unethical conduct by their employees, a preliminary ANOVA was implemented to exclude mean differences. Furthermore, since some clinical misbehavior and CWB-P items were extremely skewed we treated them as categorical and used accordingly WLSMV (mean- and variance-adjusted weighted least squares) as parameters estimation method in Mplus.

### Results

#### Descriptive Statistics

As shown in **Table 2**, all measures have good reliability. Furthermore, the correlations were all significant. Results of a preliminary ANOVA confirmed that there were not mean differences among the six hospitals in relation to the levels of both clinical misbehavior (*F*_5,410_ = 1.543, *p* = 0.175) and CWB-P (*F*_5,410_ = 1.357, *p* = 0.240). This result was further confirmed by a non-significant result for the ANOVA in relation to MD (*F*_5,410_ = 1.730, *p* = 0.126).

**Table 2:** Study 2: descriptive statistics among study variables.

	*M*	*SD*	Skewness	Kurtosis	Cronbach’s alpha	2	2a	2b	2c	3	4	5	6
(1) Third-party aggression	2.09	0.99	0.95	0.17	0.84	0.34^∗∗^	0.31^∗∗^	0.28^∗∗^	0.32^∗∗^	0.15^∗∗^	0.14^∗∗^	0.17^∗∗^	0.25^∗∗^
(2) Negative emotions	2.32	0.76	0.61	-0.02	0.90	–	0.89^∗∗^	0.77^∗∗^	0.88^∗∗^	0.33^∗∗^	0.34^∗∗^	0.36^∗∗^	0.55^∗∗^
(2a) Anger	2.49	0.96	0.76	0.18	0.83		–	0.61^∗∗^	0.70^∗∗^	0.23^∗∗^	0.24^∗∗^	0.25^∗∗^	0.46^∗∗^
(2b) Fear	2.08	0.86	0.40	-0.36	0.70			–	0.57^∗∗^	0.31^∗∗^	0.37^∗∗^	0.36^∗∗^	0.42^∗∗^
(2c) Sadness	2.22	0.92	0.67	-0.15	0.70				–	0.26^∗∗^	0.27^∗∗^	0.28^∗∗^	0.46^∗∗^
(3) Moral disengagement	1.44	0.52	2.70	9.47	0.90					–	0.42^∗∗^	0.39^∗∗^	0.11^∗^
(4) Clinical deviant behavior	1.39	0.61	2.69	9.03	0.82						–	0.84^∗∗^	0.14^∗∗^
(5) CWB-P	1.41	0.71	2.25	5.24	0.86							–	0.17^∗∗^
(6) Health symptoms	2.64	0.77	-0.21	-0.66	0.86								–


**CWB-P, interpersonal counterproductive work behavior; ^∗^p < 0.05, ^∗∗^p < 0.01.**

##### Model 1 – General negative emotion

The measurement model fits the data well: χ^2^(176) = 276.04, *p* < 0.01; *CFI* = 0.98; *RMSEA* = 0.037 (*90% C.I.* = 0.028-0.045), *p* = 1.00; WRMR = 1.161. Results of the one-factor model showed a poor fit to the data: χ^2^(189) = 519.06, *p* < 0.001, *CFI* = 0.93, *RMSEA* = 0.065 (95% *C.I.* = 0.058–0.071), *p* = 0.001; *WRMR* = 2.624 attesting to the discriminant validity of the measures as also confirmed by the significant chi-square difference test between the two models (*p* < 0.001).

**FIGURE 4:**
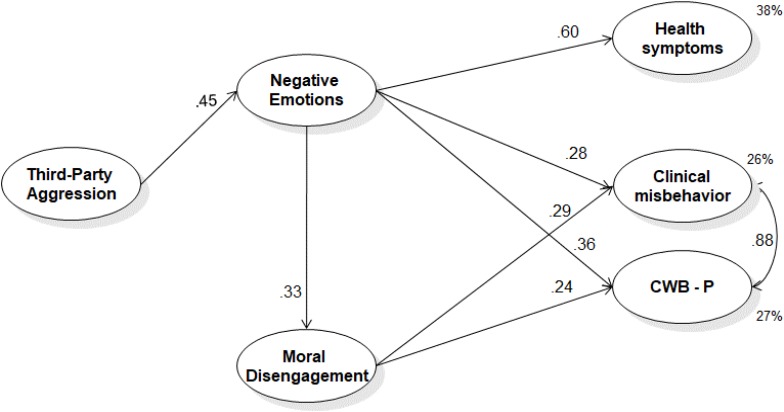
Study 2: results of the posited Model 1 – general negative emotion. All variables are latent variables measured by their indicators as described in the text. Completely standardized robust maximum likelihood parameter estimates. All coefficients reported are significant for *p* < 0.05. The results of the role of the covariates and the non-significant hypothesized paths have not been reported in the figure, but they are discussed in the text. CWB-*P*, interpersonal counterproductive work behavior.

Model 1, presented in **Figure 4**, shows a satisfactory fit [χ^2^(213) = 310.40, *p* < 0.01; *CFI* = 0.97, *RMSEA* = 0.033 (*90% C.I.*: 0.025-0.041), *p* = 1.00; *WRMR* = 1.207] and confirmed all the hypothesized paths and indirect effects (H4, β = 0.27, *p* < 0.01; H5, β_clinical_ = 0.13, *p* < 0.01, β_CWB-__P_ = 0.16, *p* < 0.01; H8, β_clinical_ = 0.04, *p* < 0.01, β_CWB-__P_ = 0.04, *p* < 0.01).

##### Model 2 – Discrete negative emotions

The measurement model resulted in a good fit: χ^2^(178) = 273.45, *p* < 0.01; *CFI* = 0.98; *RMSEA* = 0.036 (*90% C.I.* = 0.027-0.044), *p* = 1.00; *WRMR* = 1.164. Results of the one-factor model showed a poor fit to the data: χ^2^(189) = 503.21, *p* < 0.001, *CFI* = 0.94, *RMSEA* = 0.063 (*90% C.I.* = 0.057-0.070), *p* = 0.001; *WRMR* = 2.608, attesting to the discriminant validity of the measures and the absence of common method bias. This result is also confirmed by the significant chi-square difference test between the two models (*p* < 0.001).

**FIGURE 5:**
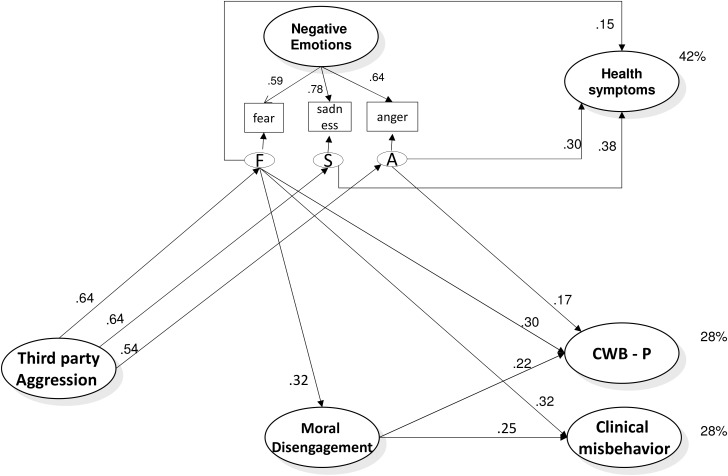
Study 2: results of the posited Model 2 – discrete negative emotions. All variables are latent variables measured by their indicators as described in the text. Completely standardized robust maximum likelihood parameter estimates. All coefficients reported are significant for *p* < 0.05. The results of the role of the covariates and the non-significant hypothesized paths have not been reported in the figure, but they are discussed in the text. F, residual variance of Fear, that is, what Fear does not share with Anger and Sadness; S, residual variance of Sadness, that is, what Sadness does not share with Anger and Fear; A, residual variance of Anger, that is, what Anger does not share with Fear and Sadness; CWB-*P*, interpersonal counterproductive work behavior.

Model 2, summarized in **Figure 5**, resulted in a good fit [χ^2^(203) = 321.96, *p* < 0.01; *CFI* = 0.98; *RMSEA* = 0.038 (*90% C.I.* = 0.030-0.045), *p* = 1.00; *WRMR* = 1.208] and fully confirmed all the hypotheses with the exception of H3 and H7. In particular, anger was associated only with CWB-P but not with clinical misbehavior (H3a). In addition, only fear but not anger was associated with MD (H7a and H7b). Indirect effects were also confirmed (H4a, β = 0.16, *p* < 0.01, H4b, β = 0.10, *p* < 0.01, H4c, β = 0.25, *p* < 0.001, H5b, β = 0.20, *p* < 0.01, H5b, β = 0.19, *p* < 0.05, H5a, β = 0.09, *p* < 0.01, H8b, β_clinical_ = 0.06, *p* < 0.01, β_CWB-__P_ = 0.05, *p* < 0.01).

### Discussion

Results of this study showed that third-party aggression could be associated not only with targets’ health symptoms but also with engaging in misbehavior. Specifically, results from Model 1 confirm the hypotheses that the negative emotions experienced when being target of mistreatment by patients and their relatives are associated with CWB directly, and indirectly through MD. However, when examining separately anger, fear, and sadness results showed that while sadness was only associated with health symptoms, anger and fear were also associated with CWB-P and clinical misbehavior, although in a different way. Indeed, anger was associated only with CWB-P directly, without the mediation of MD. Fear was associated with both CWB-P and clinical misbehavior both directly and indirectly through MD.

## General Discussion

Results from our two empirical studies confirm the hypotheses that being target of workplace aggression (bullying or third-party aggression) is not only associated with health symptoms but also with misbehavior. This research underlines, in line with Kong and Drew (2016), the relevance of examining concurrently different discrete negative emotions and MD. In particular, this research shows for the first time that anger, fear, and sadness, generally aggregated into a single dimension, are indeed differently associated with MD, misbehavior and health symptoms. In line with the literature on discrete emotions, while sadness is only associated with health symptoms, anger and fear are related to both health and misbehavior.

Findings from both studies, consistent with a large body of research, confirm that workplace aggression is associated with negative emotions either when considering an overall indicator or when considering anger, fear, and sadness separately. Indeed, although workplace bullying and third-party aggression differ in terms of sources, dynamics, and type of aggression, in both cases targets potentially experience all these three specific emotions.

As pertaining to the specific roles of the three discrete emotions the main findings, cross-validated in both studies, are: anger is associated with the engagement in specific forms of CWB, although contrary to our expectation only directly; fear is associated with the engagement in CWB mainly through the mediation of MD; sadness is not significantly associated with CWB, being only related to physical symptoms (as anger and fear as well).

Both studies provide support to the literature suggesting that being a target of aggression represents a frustrating situation in which targets experience anger that may prompt a ‘hot’ and impulsive aggressive response (Averill, 1982). Indeed, consistent with models of displaced aggression (Berkowitz, 1989) and the excitation transfer paradigm, it is likely that targets of aggression could dislocate the anger activation to others individuals in other contexts. This direct path agrees with literature according to which the anger activation resulting from workplace aggression may direct the target of aggression to find a ‘way out’ (Averill, 1982), and it is likely that the ‘form of expression’ the target may use mirrors the behavioral models to which they were exposed (Huesmann and Kirwil, 2007). In addition, the perceived damage to individual’s identity and the plausible perceived violations of one’s own rights – possibly associated with being a target of aggression – may increase a hostile mind-set, predisposing targets to act aggressively (Anderson and Bushman, 2002). It must be acknowledged that the direct association of anger with CWB is confirmed only for CWB-P but not for both CWB-O and clinical misbehavior. CWB-P comprises mainly a range of mild aggressive behaviors, such as insulting or making an obscene gesture, which may have a prevalently impulsive connotation. In contrast, both CWB-O, as assessed in Study 1, and clinical misbehavior, as assessed in Study 2, include forms of misconduct violating explicit organizational rules and protocols and as such it may be likely that anger may be controlled without finding a ‘way out’ in these types of behavior.

Contrary to our expectations the association between anger and CWB is not mediated by MD. In line with the broad literature on MD, our results confirm its disinhibitory power, and its association with both aggressive behavior within interpersonal interactions (CWB-P) and deviant conduct (CWB-O and clinical misbehavior). Specifically, findings suggest that nurses may rely on MD to preserve their moral standards when misbehaving. Notwithstanding this, anger seems to trigger impulsive behaviors which do not necessary required the activation of MD to provide a justification or a legitimization. The lack of cognitive mediation may be explained considering the psychological functioning characterizing anger and the plausible impulsive nature of the aggressive behavior. Anger is’ experienced when an event is perceived as unfair, hence individuals believe to be on ‘the right side’ and they may not need a justification for engaging in an aggressive behavior without the intention of violating explicit professionals norms.

This research provides evidence in both studies, for the first time in the literature, of fear being an important discrete emotion associated with CWB through MD. Indeed, although Bandura (2004) suggested the potential relevance of fear, this association was not previously empirically tested. Since individuals experiencing fear are more alert and attentive to pick up potential external threats, and tend to perceive the environment as highly dangerous and threatening (Öhman, 2008), they are more likely to engage in any form of behavior that may potentially help them to defend themselves, including aggression (Simunovic et al., 2013). Hence, MD may come into play in this process, facilitating the justification of potential defensive (even preventive) misbehaviors. In other words, fear may lead target of aggression to engage in self-serving mechanisms that may lead to CWB to defend and comply with their need for protection.

In addition, it must be acknowledged that fear was also directly associated with both CWB-P and clinical misbehavior, but only in Study 2. This finding may be explained considering the specific nature of third-party aggression. Indeed, in Study 2 workplace aggression refers to direct aggressive acts putting potentially at risk targets’ physical safety. Hence, this may result in making targets more sensitive to threatening events and, consequently, to be more incline to engage in misbehavior as a way to defend themselves.

Finally, as expected, findings confirm that sadness is not associated with CWB, neither directly nor indirectly, but it is exclusively associated with health symptoms. This possibly suggests that the experience of the loss and the inward focus may lead to a set of symptoms that are functional to the ‘time-out’ state typical of sadness. Similarly, individuals who frequently experience this emotion tend to pay a greater attention to their symptoms – which are then reported more frequently – possibly as a help seeking strategy (cf. Consedine et al., 2006). Furthermore, it must be acknowledged that fear and anger are also associated with health symptoms, in line with the existing literature (e.g., Consedine and Moskowitz, 2007). Hence overall, results of this research confirm that the emotional experience associated with being target of aggression (bullying or third party aggression) is associated with a range of health symptoms affecting nurses’ well-being.

### Limitations and Future Studies

The cross-sectional nature of our data makes it more difficult for us to infer causal relationships among the variables considered, although the proposed model is strongly based on prior theories and it was possible to replicate largely it in two independent samples. Future longitudinal and diary studies could be designed and implemented to strengthen further the model and test potential reciprocal relationships. In addition, it is plausible that other variables may intervene in the process examined in the research and it would be worthy to explore more in depth the interpretation that individuals provide to the different aggressive events they experience. To this end, qualitative and diary study may be particularly helpful to further disentangle and understand the interplay between emotions and cognitive processes.

Future studies should also broaden the range of discrete emotions considering for example guilt, shame and embarrassment. In addition, the role of social and organizational factors should also be investigated. Another limitation of this research is the lack of specific information about the sources of workplace bullying and about the specific target of CWB-P. As a consequence, it was not possible to examine whether the different sources of aggression have any association with the specific recipient of CWB-P. Finally, another limitation of this research is the use of self-report instruments particularly when considering the illegal nature of some of the CWB measured that may result in underreporting. However, it should be noted that Fox et al. (2007) demonstrated the convergence between self- and peer-reports CWB measures.

### Practical Implications

In healthcare services, the prevention of aggression and violence and their negative consequences is still a challenge for many scholars and professionals (Arnetz et al., 2017). Our research can have relevant implications for healthcare decision makers. Overall, the results of our study offer suggestions for designing prevention programs aimed at increasing employees’ well-being, the quality of the interactions with patients and staff, and, in general, the quality of care.

Although the relevance of emotions for both targets and perpetrators of aggression has been extensively highlighted in the literature, in some of the most recent and popular guidelines for preventing workplace violence (e.g., International Labour Organization [ILO], 2005; Centers for Disease Control and Prevention, 2006; OSHA, 2015) there is no specific focus on emotions. For example, the Centers for Disease Control and Prevention (National Institute for Occupational Safety and Health [NIOSH], 2002) suggests including administrative, behavioral and environmental strategies for violence reduction, but, in their suggestions for training programs emotional processes are not included. In the OSHA’s (2015, p. 25) guidelines it is written that ‘training topics may include management of assaultive behavior, professional/police assault-response training, or personal safety training on how to prevent and avoid assaults.’ Our findings, in line with the literature on emotions and aggression (Grandey et al., 2004), suggest that training programs should also focus on emotions and in particular on the specificity of the emotional experience. For example, training should help employees to gain awareness about the different possible emotional responses associated with the experience of aggression at work that may potentially lead to different dysfunctional paths for themselves and the organization stakeholders. In addition, trainings should be designed taking into account and working accordingly on the different processes associated with specific emotions experienced by targets of aggression. For instance, sessions should be focused on de-escalating processes to address anger response, while focused on perceived control to address fear response.

The development of emotional regulation skills may also help to reduce the risk of activating justification cognitive processes as MD, especially when targets of aggression experience fear. Indeed, according to our results, fear is associated with this type of mechanism that allows individuals to deactivate their internal moral control and engage in misbehaviors. In relation to the relevance of MD, it is also important to design and implement interventions aimed at promoting an ethical culture. For instance, the presence of ethical models may not only directly reduce workplace aggression and MD by making the ethical norms and practice more salient, but it may also provide examples of strategies to deal with threatening and hostile interactions.

## Conclusion

In summary, workplace aggression is a relevant phenomenon with potential consequences, not only for the direct victim, but also for the entire organizational system, in which it is possible to envision the trigger of vicious circles leading to broader and more diffuse forms of workplace aggression. The present research suggests the importance of examining affective and cognitive processes that could mediate the relationship between experienced aggression and misbehaving. In particular, findings suggest that the experience of anger and fear associated with being the target of aggression at work could lead some nurses to translate their emotional activation into misconduct possibly disregarding professional and ethical codes.

In addition, this study highlights the importance of studying MD that could facilitate the spread of misbehavior across different situations. As suggested by Bandura (2002), MD can explain the part of the process through which misbehavior can become routine. In particular, he stated that the ‘continuing interplay between moral thought, affect, action and its social reception is personally transformative. People may not even recognize the changes they have undergone as a moral self’ (Bandura, 2002, p. 110).

## Ethics Statement

This study was carried out in accordance with the Sapienza University of Rome ethical guidelines and approved by the Research Ethics Committee of Sapienza University of Rome.

## Author Contributions

RF, CT, and MP substantially contributed to the conception and the design of the work, the interpretation of the data, and drafted the paper. RF was also responsible for the data collection. CG and SG contributed in the idea development and in drafting the survey whereas CB was responsible for the data analysis. CG, SG, TP, and CB reviewed the paper critically and gave important intellectual input. All authors approved the final version to be published and agreed to be accountable for all aspects of the work in ensuring that questions related to the accuracy or integrity of any part of the work are appropriately investigated and resolved.

## Conflict of Interest Statement

The authors declare that the research was conducted in the absence of any commercial or financial relationships that could be construed as a potential conflict of interest.

## APPENDIX A

### Study 2 Clinical Misbehavior Items.

Used physical restraints for agitated patients without consulting physicians.

Was superficial in updating a patient’s clinical record.

Turned off patients’ call bell during the night shift’.

Administered a therapy without consulting physicians.

Modified medical prescription [administering a higher or lower drug dose] without consulting physicians.

Adapted from Sili et al. (2014).
